# Effector-Triggered Trained Immunity: An Innate Immune Memory to Microbial Virulence Factors?

**DOI:** 10.3390/toxins14110798

**Published:** 2022-11-17

**Authors:** Cedric Torre, Laurent Boyer

**Affiliations:** Université Côte d’Azur, Inserm, C3M, 06200 Nice, France

**Keywords:** trained immunity, effector-triggered immunity, effector-triggered trained immunity

## Abstract

In the last decade, a major dogma in the field of immunology has been called into question by the identification of a cell autonomous innate immune memory. This innate immune memory (also named trained immunity) was found to be mostly carried by innate immune cells and to be characterized by an exacerbated inflammatory response with a heightened expression of proinflammatory cytokines, including TNF-α, IL-6 and IL-1β. Unlike the vast majority of cytokines, IL-1β is produced as a proform (pro-IL-1β) and requires a proteolytic cleavage to exert its biological action. This cleavage takes place mainly within complex molecular platforms named inflammasomes. These platforms are assembled upon both the infectious or sterile activation of NOD-like receptors (NLRs), thereby allowing for the recruitment and activation of caspases and the subsequent maturation of pro-IL-1β into IL-1β. The NLRP3 inflammasome has recently been implicated both in western diet-induced trained immunity, and in the detection of microbial virulence factors (effector-triggered immunity (ETI)). Here, we will attempt to link these two immune processes and provide arguments to hypothesize the existence of trained immunity triggered by microbial virulence factors (effector-triggered trained immunity (ETTI)).

## 1. Introduction

To survive host–pathogen battles, pathogens have evolved various strategies to blunt or escape host immune responses. Among them, effector proteins, also called virulence factors, could be considered as surgical weapons of microbes that precisely target the Achilles’ heel of the immune system with the goal of surviving and multiplying. Thus, the expression of virulence factors by microbes confers them with pathogenic properties, distinguishing commensals from pathogens. On the other hand, hosts have multiplied sensors to detect and gauge the pathogenic potential of microbes. Pattern Recognition Receptors (PRRs) discovery revolutionized the way of thinking about pathogen detection by linking the microbial ligand to innate immune signaling. The innate immune response subsequent to the recognition of Microbe-Associated Molecular Patterns (MAMPs) by PRR signaling has been termed Pattern-Triggered Immunity (PTI). Since this seminal discovery [1], additional sensing mechanisms have been proposed to explain how the host could distinguish commensals from pathogens.

## 2. Effector-Triggered Immunity

An elegant strategy based on the recognition of virulence factor activities was proposed. As virulence factors are specifically expressed by pathogens, being able to detect their activities appears to be a smart strategy to limit the number of sensors, which detect specifically pathogens and not all microbes. This innate immune sensing mechanism has been termed Effector-Triggered Immunity (ETI). By functioning as an additional layer of innate immune responses, ETI adds up to PTI to allow the host to gauge both the quantity of microbes and their pathogenic potential. ETI was first identified during plant–pathogen interactions, and knowledge in the matter has since deepened [2,3,4]. More recently, proofs of the conservation of this innate immune mechanism have emerged in animals [5]. Several publications have extensively reviewed the ETI similarities and differences between plants and animals [6,7,8]. Here, we will focus on the mechanisms of ETI linking RhoGTPases, inflammasomes and trained immunity.

Most bacterial toxins hijack the regulation of crucial cellular functions, such as those performed by RhoGTPases [9]. This was previously referred to as a virulence activity until recent reports showing that the modifications of RhoGTPases activity by microbial effectors are detected by the host cell through NOD-like receptors (NLRs) and the assembly of inflammasomes [5]. The Pyrin inflammasome was first shown to be activated in macrophages treated by bacterial toxins inactivating the RhoA GTPase and this finding was later extended to numerous bacterial factors inactivating the Rho family members found in various pathogenic bacteria [5,10,11,12,13]. The involvement of the NLRP3 inflammasome in ETI has recently been demonstrated, implicating the CNF1 toxin of uropathogenic *Escherichia coli* [14]. This study showed that both mouse and human macrophages detect CNF1 activity via NLRP3 inflammasome activation. CNF1 displays deamidase activity toward RhoGTPases. This modification destroys the GTPase activity and locks the RhoGTPases into an active GTP-bound form. CNF1-triggered activation of the Rac2 GTPase in macrophages activates the PAK1/2 kinases that in turn phosphorylate the Threonine 659 of human NLRP3 leading to its activation and subsequent IL-1β secretion. This way, the NLRP3 inflammasome detects the activation of Rac2 GTPase, leading to the bacterial clearance of CNF1-expressing *E. coli* in a mouse model of bacteremia. Importantly, this sensing mechanism has been reused by the innate immune system to sense other virulence factors activating Rac2 encoded by other pathogenic bacteria such as SopE from *Salmonella* spp. or DNT from *Bordetella* spp. [14].

All these studies have recently highlighted that an increasing number of virulence factors are sensed by inflammasomes; this leads to the urgent question of whether this recognition can trigger immune memory.

## 3. Trained Immunity

The immune system is classically divided into the following two compartments: the innate immunity as the first line of defense in the host’s response to aggression, and the adaptive immunity that confers immune memory to the host. The dogma that immune memory is conferred by adaptive immunity has been challenged in recent years. Moreover, the scientific literature is replete with examples of immune memory in the absence of adaptive immunity in invertebrate organisms that naturally lack adaptive immunity, a process called innate immune memory [15].

However, it was only a decade ago that these observations were made in mammals [16,17,18]. This concept of innate immune memory emerged in the early 2010s, originally referred to as an adaptive form of innate immunity before to be termed trained immunity [16,19,20].

Mammalian innate immune memory, or Trained Immunity (TI), is a mechanism initiated by the action of an inflammatory stimulus on cells with or linked to immune functions, leading to the epigenetic and metabolic reprogramming of these cells. Thus, following a first stimulation, the prototypical cells of the innate immune compartment are trained; when restimulated, they will display an exacerbated inflammatory response that can be either beneficial or detrimental depending on the context.

Evidence of the beneficial effects for the host has been described by numerous investigations in mice [20]. As a result of these studies, it was shown that training mice with numerous microbial ligands could provide non-specific protection against an ensuing fatal infection. Beyond their specific action, live attenuated vaccines such as Bacille Calmette–Guerin (BCG), measles, smallpox or poliomyelitis vaccines have a non-specific protective action against secondary infections with other pathogens [21]. Furthermore, the BCG vaccine has proven its non-specific and beneficial action in bladder cancer, where it is used for bladder instillation treatment [22]. Conversely, immune training can lead to harmful effects, including chronic inflammatory diseases such as atherosclerotic cardiovascular disease and neurodegenerative diseases (Alzheimer disease and dementia), and tumor growth and metastasis [20].

Historically, BCG, *Candida albicans* and its β-glucan were the first triggers known to induce a TI program [17,18]; however, different types of pathological inflammation triggers were recently shown as potential TI triggers. Most of these triggers engage the activation of the IL-1 pathway (Table 1). This includes the western diet (a high fat and high sugar diet) that was shown to induce a deleterious TI program in mice and it was interestingly shown to rely on the NLRP3 inflammasome [23]. Christ and colleagues showed that feeding the western diet to *Ldlr*^−/−^ mice (a mouse model that develops atherosclerotic lesions and hypercholesterolemia) induces systemic inflammation, the proliferation of myeloid precursors, and their reprogramming, while *Ldlr*^−/−^ *Nlrp3*^−/−^ mice do not, pointing towards the importance of the NLRP3 inflammasome. Interestingly, the induction of TI by β-glucan was recently shown to limit NLRP3 inflammasome activation, repressing the production of IL-1β [24]. This highlights the importance of better characterizing the involvement of the NLRP3 inflammasome in TI. Considering that, depending on the context, NLRP3 activation triggers IL-1β secretion with or without cell death (pyroptosis versus hyperactivation), studies should be carried out in relation to inflammasome inducers and the strength of signals.

The NLRP3 inflammasome maturates both pro-IL-1β and pro-IL-18 into their biologically active forms. Interestingly, both cytokines have been associated with TI. IL-18 has been implicated in the induction of TI in specific contexts and has been associated with the involvement of TI in rheumatic pathologies such as primary Sjögren’s syndrome [25,26].

A collection of works agrees on the fact that the NLRP3-IL-1β axis is central in TI regulation, which raises the exciting possibility that TI may also be induced by the microbial effectors sensed by NLRP3 inflammasome during ETI.

**Table 1:** Evidence for the implication of the IL-1 pathway and/or NLRP3 inflammasome in trained immunity.

Model	Inducer	Challenge	Evidence for the Implication of		Reference
			IL-1 Pathway	NLRP3 Inflammasome	
Mouse	β-glucan	LPS	Yes	No	[27]
Mouse	Western diet	LPS	Yes	Yes	[23]
Mouse	β-glucan	*M. tuberculosis*	Yes	No	[28]
Mouse	Periodontitis/arthritis	LPS	Yes	Suggested	[29]
Human	BCG vaccination	*M. tuberculosis*/*S. aureus*/*C. albicans*	Yes	No	[18]
Human	BCG vaccination	LPS	Yes	No	[30]
Human	BCG vaccination	TLR ligands	Yes	No	[31]
Human	BCG vaccination	Yellow fever virus vaccine strain	Yes	Suggested	[32]
Human	BCG vaccination	TLR ligands	Suggested	No	[33]

## 4. Effector-Triggered Trained Immunity?

So far, the mechanisms of TI described in the literature involve the NOD receptor, NOD2, as well as the NLRP3 inflammasome. The NOD2 receptor has been identified as the molecular bridge between BCG vaccination and the increased production of proinflammatory cytokines, leading to the protection of severe combined immunodeficiency SCID mice from disseminated candidiasis [18]. As described earlier, the NLRP3 inflammasome is involved in TI induced by a “western diet”, a diet rich in sugar and saturated fatty acids [23]. Conversely, pyrin and NLRP3 are two major inflammasomes that have been implicated in ETI. The involvement of the NLRP3 inflammasome in both ETI and TI suggests that it could be implicated in TI in an infectious context, induced by a virulence factor, through a process that can be termed Effector-Triggered Trained Immunity (ETTI). In this view, it could be applied to bacterial effectors recently discovered for their detection by the NLRP3 inflammasome activation pathway [14]. Indeed, not only the *E. coli* CNF1 toxin but also the *Bordetella* spp. DNT (dermonecrotic toxin) and the *Salmonella* spp. SopE toxin are triggers of the NLRP3 inflammasome, suggesting their potential role as triggers of NLRP3-mediated trained immune response. If this hypothesis is true, one can expect that other NLRs involved in ETI, such as the Pyrin-inflammasome-detecting RhoA GTPase-inactivating toxins, may also be involved in ETTI. Furthermore, TI is induced by vaccination with live attenuated viruses [21]. Considering this, viruses can act as inducers of TI. Previous studies described a mechanism specific to positive single-stranded RNA ((+)ssRNA) viruses, whose proteases can induce an ETI response by activating the NLRP1 inflammasome [34]. To date, the NLRP1 inflammasome has not been implicated in TI and it would be interesting to investigate whether NLRP1 may be involved in ETTI.

The activation of the NLRP3 inflammasome requires both a transcriptional priming and an activation step triggered by various stimuli including PAMPs and DAMPs (Pathogen- and Danger-Associated Molecular Patterns). The priming step required for the production of pro-IL-1β cytokine and components for inflammasome assembly relies on the NF-κB pathway, which has been involved in the induction of TI. As an example, low-dose LPS—a well-known inducer of the NF-κB pathway—has been identified as an inducer of TI [35]. The recent study of NLRP3 activation triggered by the CNF1 toxin revealed that transcriptional priming is not mandatory to measure NLRP3-dependent Caspase-1 activation [14]. Here, we wanted to pinpoint the possible role of virulence factors in activating the inflammasome in the trained immunity process that can be observed with or without the priming step.

With regard to innate immune memory, a distinction is made between peripheral memory and central memory [20]. Peripheral memory is the memory carried by myeloid cells in circulation in the body, while central memory is the memory carried by hematopoietic stem cells in the bone marrow [36]. In this context, NLRP3 inflammasome activation has been shown to be involved in myeloid precursor release [27,37], and BCG exposure in the bone marrow modifies the transcriptional program of the hematopoietic stem and progenitor cells, promoting local cell growth and improved myelopoiesis [38]. This supports the idea of the involvement of bacterial toxins sensed by inflammasomes in the induction of a central TI.

For TI to last over time, there must be a transfer of epigenetic modifications to cells that have a long lifespan. Previously, using the planarian model, we demonstrated that an innate immune memory is carried by the neoblasts (planarian stem cells) and the innate memory could be transferred from one organism to another after irradiation; furthermore, other cells are also capable of exhibiting innate immune memory [39,40]. Later, Christ et al. proposed that the induction of a central memory within the context of the western diet is made possible by myeloid precursors [23]. Similar to these previous examples, infection with a microbe producing a microbial effector should lead to immune imprinting of myeloid precursors for a long lasting memory.

## 5. Conclusions

In this paper, we explore how trained immunity (TI) and effector-triggered immunity (ETI) are linked through the NLRP3 inflammasome, suggesting the existence of a trained immunity mediated by microbial effectors (ETTI). The involvement of the NLRP3 inflammasome in TI has been demonstrated in the context of sterile inflammation; however, its involvement in an infectious context remains to be proven. Moreover, this phenomenon might not be exclusive to the NLRP3 inflammasome and should therefore be considered for other inflammasomes. Last but not least, an interesting trans-kingdom perspective would be to return to the roots of effector-triggered immunity by determining whether trained immunity in plants is activated in response to virulence factors and whether it requires plant NLRs.

## Data Availability

Not applicable.
